# Prognostic and diagnostic significance of circRNAs expression in hepatocellular carcinoma patients: A meta‐analysis

**DOI:** 10.1002/cam4.1939

**Published:** 2019-01-28

**Authors:** Xin Huang, Weiyue Zhang, Zengwu Shao

**Affiliations:** ^1^ Department of Orthopaedics, Union Hospital, Tongji Medical College Huazhong University of Science and Technology Wuhan China; ^2^ Department of Endocrinology, Union Hospital, Tongji Medical College Huazhong University of Science and Technology Wuhan China

**Keywords:** circular RNA, diagnosis, hepatocellular carcinoma, meta‐analysis, prognosis

## Abstract

Circular RNAs (circRNAs) as novel biomarkers are widely investigated in various cancers. The aim of our study was to reveal the clinicopathological, prognostic, and diagnostic features of circRNAs in human hepatocellular carcinoma (HCC). A systematical search was conducted on PubMed, Scopus, Web of Science (WOS), EMBASE, and the Cochrane Library databases. Eligible studies reporting on the association among circRNAs and clinicopathological, prognostic, diagnostic values of HCC patients were included. Pooled odds ratios (ORs) and 95% confidence intervals (CIs) were utilized to assess clinicopathological parameters, sensitivity, and specificity, and hazard ratios (HRs) were to evaluate overall survival (OS). 17 eligible studies which included 7 for clinicopathological features, 10 for prognosis, and 8 for diagnosis were in our study. As for clinicopathological parameters, high expression of oncogenic circRNAs had a significant association with poor clinicopathological features and tumor‐suppressor circRNAs proved the contrary. In terms of the prognostic values, oncogenic circRNAs had a negative influence on overall survival (OS: HR = 3.39, 95%Cl: 2.59‐4.19), and high expression of tumor‐suppressor circRNAs was relevant to improved survival outcomes (OS: HR = 0.46, 95%Cl: 0.37‐0.56). The pooled diagnostic outcomes indicated an area under the curve (AUC) of 0.84, with sensitivity of 82% and specificity of 72% in discriminating HCC from controls. Our study indicates that circRNAs may be important biomarkers for prognostic and diagnostic values of HCC.

## 1 INTRODUCTION

As a novel class of endogenousnoncoding RNA, circular RNA (circRNA) generates from the back splicing by the canonical spliceosome.^1^ Rather than the line structure with a 5’ cap or a 3’ polyadenylated tail, circRNAs are characterized by a covalently closed loop structure.^2^, ^3^ Because of the stable and conserved characteristics, circRNAs are supposed to become required novel indicators and therapeutic targets for human cancers.^4^, ^5^ Moreover, circRNAs might be particularly expressed in a special cell line or developmental stage.^6^ The growing number of articles suggests that numerous functions of circRNAs including regulating transcription process and RNA splicing, functioning as microRNA sponges, and translation into different proteins which was processed by N6‐methyladenosine (m6A) modification.^7^, ^8^ However, more underlying mechanisms and functions of circRNAs remain largely unknown.

CircRNAs have been recently confirmed to have regulative functions in tumorigenesis, development of cardiovascular problems, and pathogenesis of neurodegenerative diseases,^9^ whereas the abnormal expression level and various functions of circRNAs in human hepatocellular carcinoma (HCC) remain largely unknown. HCC is the most common primary malignancy of the liver^10^ and one of the major causes of cancer‐related death worldwide, especially in China.^11^ For early‐stage HCC patients, surgical treatments including liver resection and transplantation are still the most curative treatment methods. Whereas taken the high incidence of postoperative recurrence into account, the prognosis after curative resection of HCC has remained unsatisfactory.^12^

In our study, we performed a meta‐analysis to summarize the clinicopathological, prognostic, and diagnostic significances of circRNAs in HCC patients. Further prospective studies including more kinds of circRNAs are warranted in the future.

## 2 MATERIALS AND METHODS

### 2.1 Data search strategy

A computerized literature search was performed in the PubMed, Scopus, Web of Science (WOS), EMBASE, and the Cochrane Library databases up to 17 July 2018. The search strategy of our study followed the terms such as: (a) “circRNA” or “circular RNA”; and (b) “liver cancer” or “liver carcinoma” or “liver tumor” or “hepatocellular carcinoma” or “HCC.” Additionally, we hand‐searched the references of all relevant articles one by one if it is necessary. When the important data were not available, we tried to contact researchers of certain articles.

### 2.2 Inclusion and exclusion criteria

The article selection used the following inclusion and exclusion criteria for each study.

A study that is eligible for inclusion must meet the following criteria:

1. Case‐control study or cohort study including both case and control groups.
2. Patients with a pathological diagnosis of HCC.
3. Detection of circRNA expression level, clinicopathological features, and prognosis of patients.

Moreover, the exclusion articles all fitted the following criteria:

1. Studies not relevant to circRNA or HCC.
2. Similar studies or duplicate data in the different articles.
3. Animal studies, reviews, case serious, expert opinions, letters.
4. Without available data for analysis and the authors could not be contacted.
5. Not English language.

### 2.3 Data extraction and quality assessment

Two investigators (Xin Huang and Weiyue Zhang) were assigned to assess the eligibility of all studies, and the relevant data for analysis were extracted on their own. Moreover, a third investigator (Zengwu Shao) resolved the disagreements when necessary. The important data were collected as follows: (a) baseline information of each study including author, year of publication, circRNA type, cancer type, case number, and detection method; (b) we extracted data involving upregulated and downregulated expression role of circRNAs, duration of follow‐up, and overall survival (OS) for prognosis analysis; (c) in diagnostic analysis, the sensitivity, specificity, and an area under the curve (AUC) were also collected; and (d) clinicopathological information including age, gender, tumor size, TNM stage, differentiation, vascular invasion, liver cirrhosis, lymphatic metastasis, distant metastasis, and so on. The study quality was assessed in accordance with the Newcastle‐Ottawa Scale (NOS) (Table S1). The study was considered high quality with the scores were ≥7. Our study was in keeping with the Preferred Reporting Items for Systematic Reviews and Meta‐Analyses (PRISMA) statement.

### 2.4 Statistical analysis

The statistical data were analyzed by Stata version 14.0. Pooled odds ratios (ORs) and 95% confidence intervals (CIs) wereutilized to evaluate clinicopathological parameters, sensitivity, and specificity, and hazard ratios (HRs) were to evaluate overall survival (OS). The chi‐square test and the *I* ^2^ statistic were utilized to assess the between‐study heterogeneity. If an *I* ^2^ value of <50%, it was considered that no significant heterogeneity existed.^13^ A random‐effects model was utilized when there was a significant heterogeneity. Otherwise, the fixed‐effects model was used.^14^ We further made sensitivity analyses to detect the stability of results and the potential source of heterogeneity.^14^ Begg, Egger, and Deeks’ tests were mainly used to assess the publication bias.^15^

## 3 RESULTS

### 3.1 Search results

**Figure 1:**
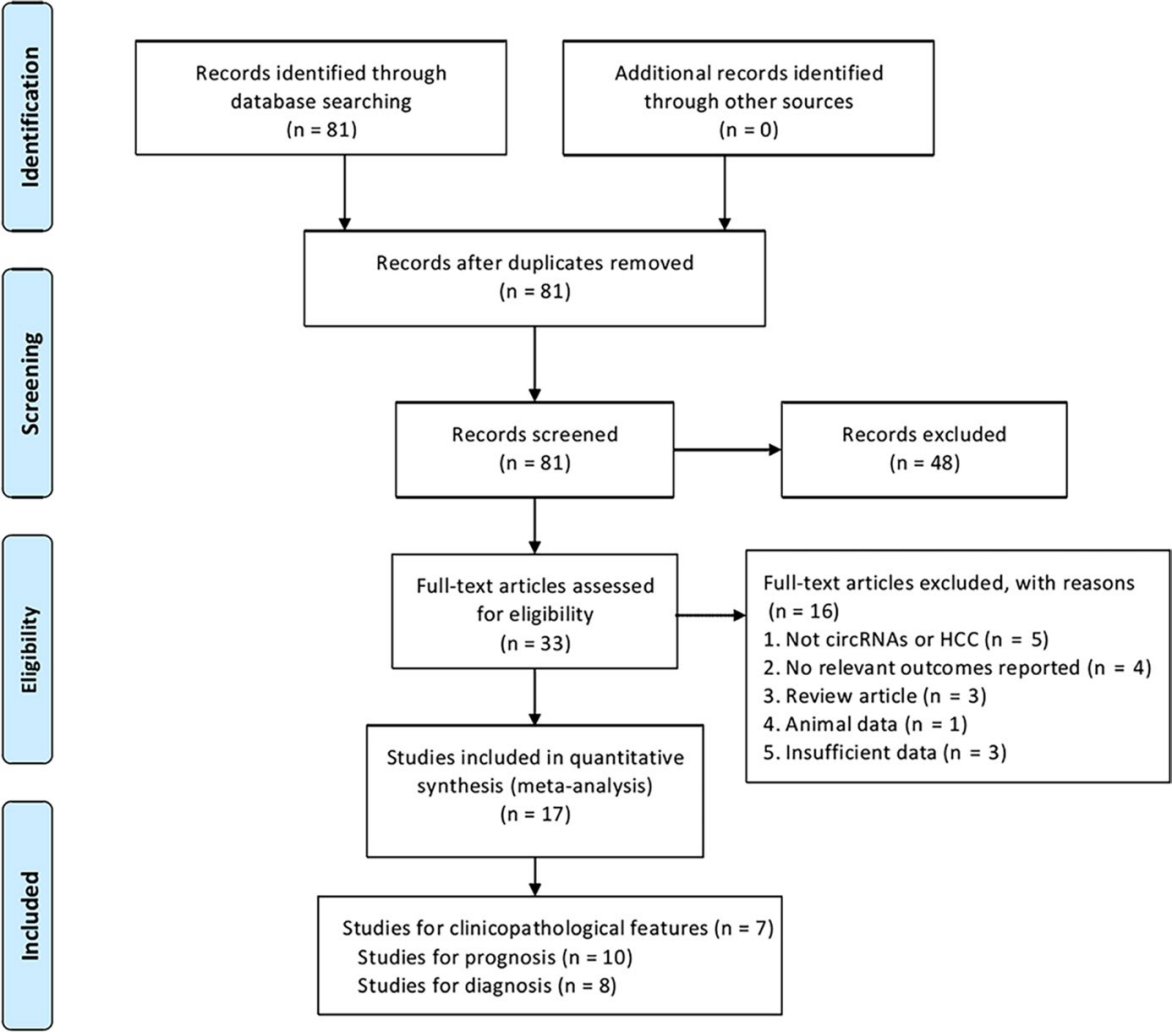
Flowchart of the study selection process

The study search is shown in the flow diagram (Figure 1). 81 relevant articles were collected during the database search. Furthermore, 48 were eliminated after abstract review, leaving 33 articles for further review. During the full‐text review, 16 studies were eliminated for the following reasons: 5 were not associated with circRNAs or HCC, 4 were without relevant data included, 3 were reviews, 1 was animal experiments, and 3 were of insufficient data for analysis. To sum up, 17 eligible studies with 1798 HCC patients were included in the present study. All the selected studies included 7 for clinicopathological features, 10 for prognostic analysis, and 8 for diagnostic analysis.

### 3.2 Study characteristics

The main features of each eligible study are summarized in details (Tables 1 and 2). The years for publication ranged from 2017 to 2018. The range of sample size in each study was from 47 to 288. Moreover, the quantitative real‐time polymerase chain reaction (qRT‐PCR) was used to measure circRNA expression levels. As shown in Table 1, six types of circRNAs were recognized as tumor promoters and five were tumor suppressors. The range of mean duration of follow‐up was from 30 to 118 months. Moreover, the sensitivity, specificity, and AUC were extracted from eight studies for diagnosis analysis (Table 2). According to the Newcastle‐Ottawa Scale (NOS), the quality scores of all included studies ranged from 7 to 8, which indicated a high quality (Table S1).

**Table 1:** Main characteristics of studies for prognosis analysis

Study	Year	CircRNA	Cancer type	CircRNA expression		Detection method	Regulation	Follow‐up (mo)	Citation
				High	Low				
Xu et al	2017	circCdr1as	HCC	48	47	qRT‐PCR	Upregulated	62	16
Meng et al	2018	circ_10720	HCC	32	65	qRT‐PCR	Upregulated	118	17
Zhu et al	2018	circ_0067934	HCC	25	25	qRT‐PCR	Upregulated	60	18
Chen et al	2018	circ_0128298	HCC	39	39	qRT‐PCR	Upregulated	66	19
Huang et al	2017	circ_100338	HCC	29	51	qRT‐PCR	Upregulated	115	20
Zhang et al	2018	circSMAD2	HCC	43	43	qRT‐PCR	Upregulated	30	21
Han et al	2017	circMTO1	HCC	116	116	qRT‐PCR	Downregulated	80	22
Zhang et al	2018	circ_0001649	HCC	35	42	qRT‐PCR	Downregulated	44	23
Guo et al	2017	circ‐ITCH	HCC	100	188	qRT‐PCR	Downregulated	83	24
Zhong et al	2018	circC3P1	HCC	24	23	qRT‐PCR	Downregulated	60	25
Yu et al	2018	circ cSMARCA5	HCC	78	85	qRT‐PCR	Downregulated	60	26

HCC, hepatocellular carcinoma; qRT‐PCR, quantitative real‐time polymerase chain reaction.

**Table 2:** Main characteristics of studies for diagnosis analysis

Study	Year	CircRNA	Cancer type	Sample size		Method	Regulation	Diagnostic power			Citation
				Case	Control			Citation	Spe	AUC	
Xu et al	2017	circCdr1as	HCC	48	47	qRT‐PCR	Upregulated	0.753	0.669	0.680	16
Chen et al (1)	2018	circ_0128298	HCC	39	39	qRT‐PCR	Upregulated	0.906	0.553	0.664	19
Chen et al (2)	2018	circ_0091582	HCC	39	39	qRT‐PCR	Upregulated	0.782	0.685	0.679	19
Chen et al (3)	2018	circ_0091528	HCC	39	39	qRT‐PCR	Upregulated	0.754	0.581	0.601	19
Guan et al	2017	circ_0016788	HCC	40	40	qRT‐PCR	Upregulated	0.923	0.756	0.851	27
Zhang and Zhou	2018	circ_0001445	HCC	55	44	qRT‐PCR	Downregulated	0.926	0.801	0.862	28
Fu et al	2017	circ_0004018	HCC	102	102	qRT‐PCR	Upregulated	0.716	0.815	0.848	29
Yao et al	2017	circZKSCAN1	HCC	102	102	qRT‐PCR	Downregulated	0.822	0.724	0.834	30
Shang et al	2016	circ_0005075	HCC	30	30	qRT‐PCR	Upregulated	0.833	0.900	0.940	31
Qin et al	2016	circ_0001649	HCC	89	89	qRT‐PCR	Downregulated	0.810	0.695	0.631	32

AUC, area under the ROC curve; HCC, hepatocellular carcinoma; qRT‐PCR, quantitative real‐time polymerase chain reaction; Sen, sensitivity; Spe, specificity.

### 3.3 Meta‐analysis for clinicopathological parameters

At first, we evaluated the relationship between circRNAs and clinicopathological parameters of HCC (Table 3). A significant association between high expression of oncogenic circRNAs and poor clinicopathological characteristics (tumor size: OR = 1.82, 95%Cl: 1.39‐2.38; TNM stage: OR = 2.07, 95%Cl: 1.51‐2.84; differentiation grade: OR = 1.89, 95%Cl: 1.48‐2.42; vascular invasion: OR = 1.83, 95%Cl: 1.34‐2.53; liver cirrhosis: OR = 1.52, 95%Cl: 1.17‐1.97; lymph node metastasis: OR = 2.83, 95%Cl: 1.77‐4.52; distant metastasis: OR = 3.50, 95%Cl: 1.51‐8.12; serum AFP: OR = 2.07, 95%Cl: 1.63‐2.64; HbsAg‐positive: OR = 1.65, 95%Cl: 1.24‐2.22) was observed in our study. Furthermore, our meta‐analysis showed that high expression of tumor‐suppressor circRNAs was significantly associated with better clinical parameters (tumor size: OR = 0.56, 95%Cl: 0.33‐0.96; TNM stage: OR = 0.68, 95%Cl: 0.58‐0.81; differentiation grade: OR = 0.69, 95%Cl: 0.56‐0.85; vascular invasion: OR = 0.48, 95%Cl: 0.32‐0.74), whereas there were no significant relationships between high tumor‐suppressor circRNAs expression and other clinicopathological parameters including age, gender, liver cirrhosis, serum AFP, and HbsAg‐positive.

**Table 3:** Clinical characteristics of circRNAs in HCC

	Tumor promoter			Tumor suppressor		
	OR	95% Cl	*P*	OR	95% Cl	*P*
Age	1.162	0.879‐1.537	0.291	0.973	0.732‐1.292	0.849
Gender (M/W)	0.921	0.790‐1.073	0.289	0.967	0.846‐1.104	0.615
Tumor size	**1.818**	**1.385‐2.387**	**0.000**	**0.564**	**0.331‐0.960**	**0.035**
TNM stage (III + IV/I + II)	**2.073**	**1.512‐2.842**	**0.000**	**0.686**	**0.579‐0.811**	**0.000**
Differentiation grade	**1.891**	**1.476‐2.422**	**0.000**	**0.691**	**0.559‐0.854**	**0.001**
Vascular invasion (Y/N)	**1.837**	**1.335‐2.529**	**0.000**	**0.489**	**0.324‐0.738**	**0.001**
Liver cirrhosis (Y/N)	**1.515**	**1.167‐1.965**	**0.002**	1.071	0.879‐1.305	0.497
Lymph node metastasis (Y/N)	**2.830**	**1.773‐4.516**	**0.000**	NA	NA	NA
Distant metastasis (Y/N)	**3.500**	**1.509‐8.116**	**0.004**	NA	NA	NA
Serum AFP	**2.071**	**1.627‐2.637**	**0.000**	0.958	0.784‐1.172	0.678
HbsAg (P/N)	**1.658**	**1.235‐2.226**	**0.001**	1.056	0.900‐1.240	0.503

The results are in bold if *P* < 0.05.

CI, confidence interval; M, men; N, no/negative; NA, not available; OR, odds ratio; P, positive; W, women; Y, yes.

### 3.4 Meta‐analysis for overall survival

**Figure 2:**
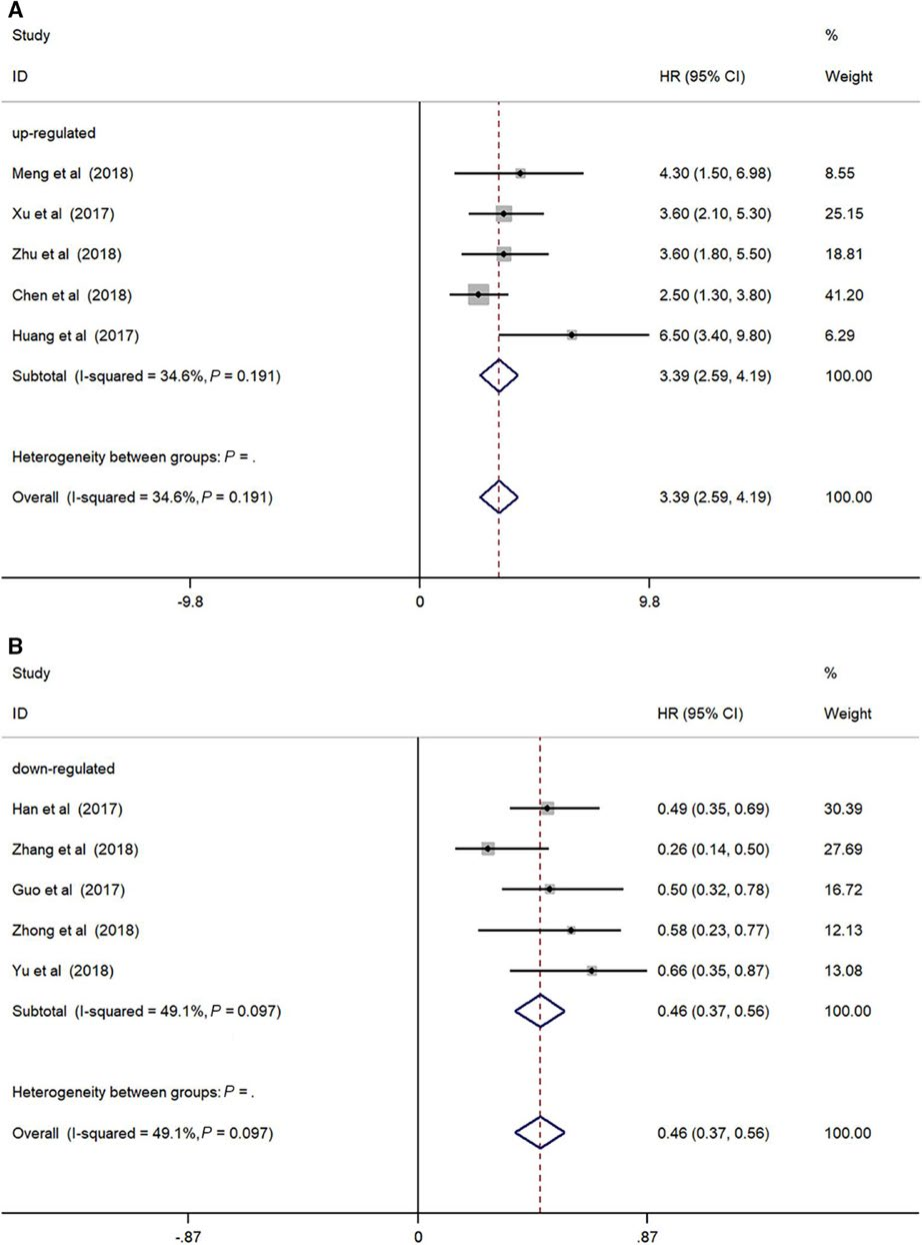
Forest plots for overall survival (OS) according to the type of (A) oncogenic circRNAs and (B) tumor‐suppressor circRNAs in HCC patients

As shown in Figure 2A, oncogenic circRNAs overexpression was significantly correlated with a worse prognosis (OS: HR = 3.39, 95%Cl: 2.59‐4.19, *P* < 0.001), and we adopted the fixed‐effect model because of no significant heterogeneity (*I* ^2^ = 34.6%, *P* = 0.191). Additionally, our study indicated that tumor‐suppressor circRNAs overexpression was related with improved survival (OS: HR = 0.46, 95%Cl: 0.37‐0.56, *P* < 0.001). No significant heterogeneity (*I* ^2^ = 49.1%, *P* = 0.097) was observed, and the fixed‐effect model was used (Figure 2B).

### 3.5 Meta‐analysis for diagnosis analysis

**Figure 3:**
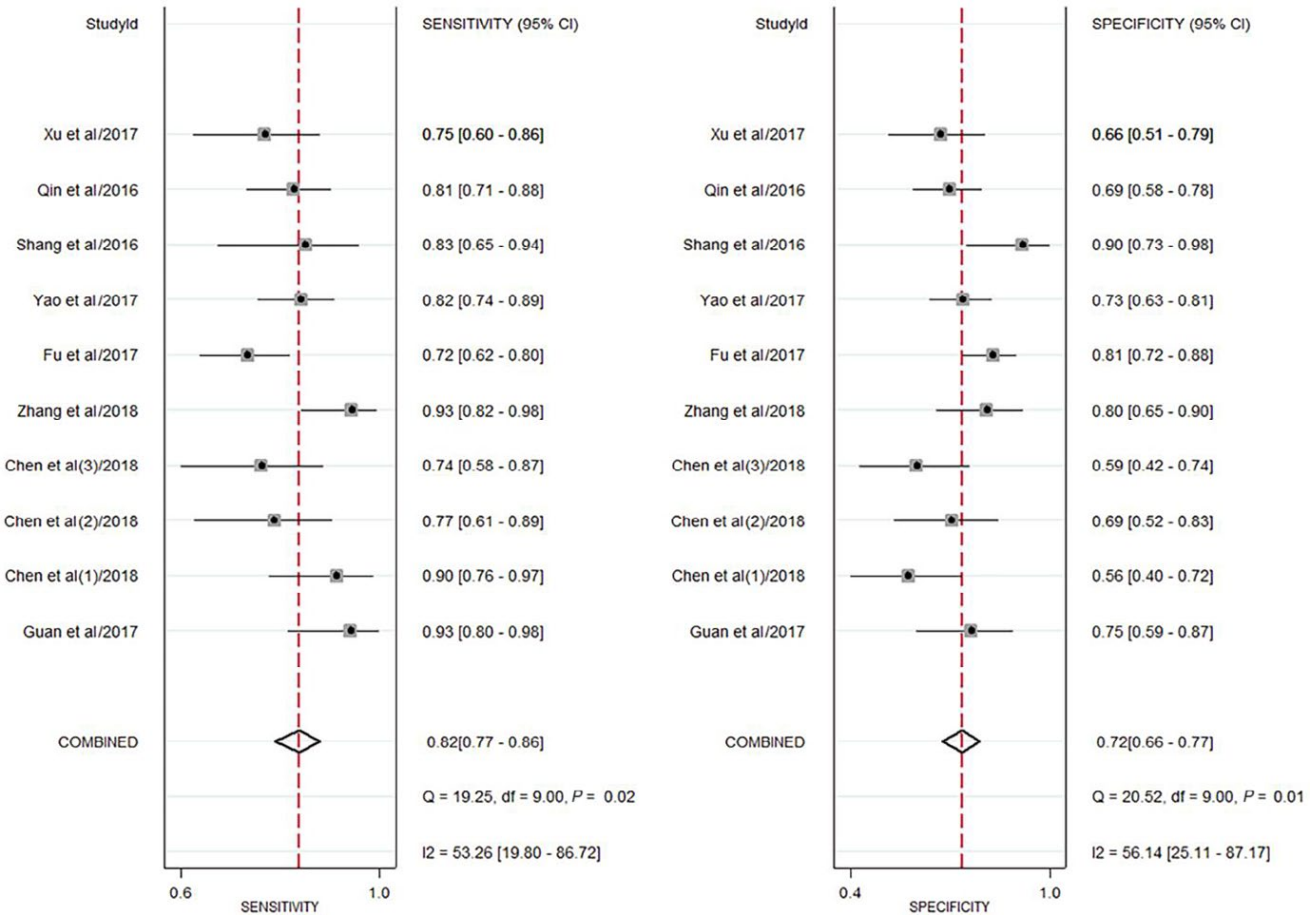
Forest plot of sensitivity and specificity of circRNAs for the diagnosis of HCC

**Figure 4:**
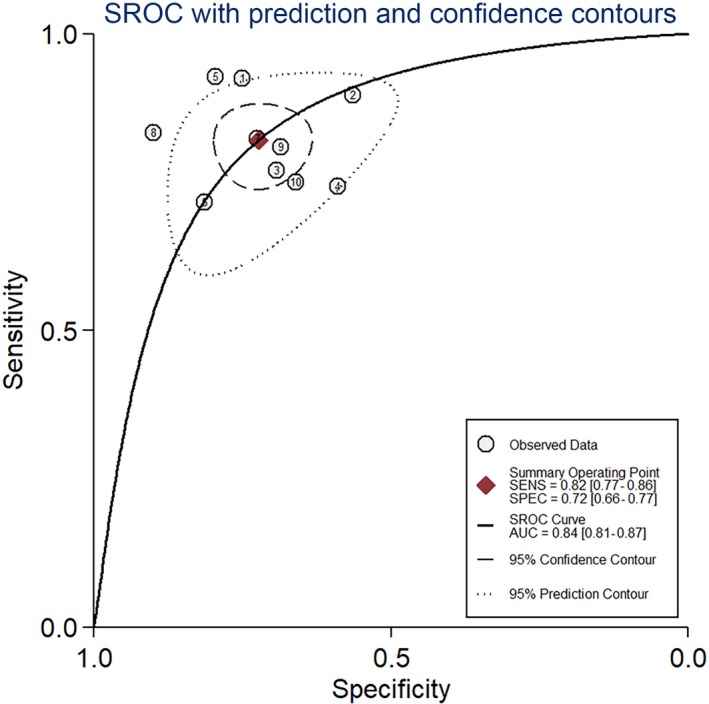
The summary receiver operator characteristic (SROC) curve

Our study showed the forest plots of sensitivity and specificity for diagnosing HCC by circRNAs (Figure 3). Because the significant heterogeneity among studies existed (*I* ^2^ = 53.3% and *I* ^2^ = 56.1%), the random‐effect model was utilized. The following pooled outcomes were sensitivity (0.82, 95%CI 0.77‐0.86) and specificity (0.72, 95%CI 0.66‐0.77). Moreover, our study performed a summary receiver operator characteristic (SROC) curve (Figure 4) and calculated AUC (0.84, 95%CI 0.81‐0.87). In summary, our study suggested that circRNAs had a good diagnostic accuracy for HCC. Further studies were warranted to verify our conclusions.

### 3.6 Publication bias and sensitivity analysis

Our study quantitatively performed Begg's and Egger's tests to assess publication bias among the eligible articles. There was no obvious publication bias according to Begg's test (*P* = 0.266) (Figure S1) and Egger's test (*P* = 0.109) (Figure S2). Therefore, we could exclude the possibility of publication bias. The sensitivity analysis revealed that the main outcomes of our study did not alter greatly when deleting studies one by one (Figure S3). We conducted a Deeks’ funnel plot asymmetry test^33^ with no obvious publication bias (*P* = 0.53) observed for diagnostic studies (Figure S4).

## 4 DISCUSSION

The pivotal role of circRNAs in cancers was widely acknowledged in recent studies, whereas no relevant meta‐analysis on circRNAs expression in HCC existed. Our study indicated a significant relationship between high expression of circRNAs and clinicopathological, prognostic, and diagnostic significances in human HCC. Since the expression of circRNAs was upregulated or downregulated in HCC patients compared with normal tissues, we decided to recognize circRNAs as tumor promoters or tumor suppressors, respectively. 17 eligible articles including 7 for clinical parameters, 10 for prognosis, and 8 for diagnosis were included in our study. For clinicopathological features, oncogenic circRNAs overexpression was significantly related with worse clinicopathological characteristics and tumor‐suppressor circRNAs proved the contrary. In terms of the prognostic values, oncogenic circRNAs had a negative influence on overall survival, and tumor‐suppressor circRNAs overexpression was associated with longer survival periods. Moreover, the summary outcomes revealed the AUC of 0.84, with sensitivity of 82% and specificity of 72% for the diagnostic values of circRNAs expression in HCC.

The diagnostic significance of circRNAs as biomarkers for HCC was firstly evaluated by our study. The results revealed that circRNAs are appropriate as diagnostic biomarkers for HCC. Because the dysregulated expressions of circRNAs were detected successfully in cancer cells, tumor tissues, and even plasma samples from patients, it was convenient and inexpensive for us to get samples and have an examination. Moreover, the conserved and stable structures of circRNAs enabled them to be stable under whatever circumstances. To sum up, circRNAs might be important indicators for early diagnosis of HCC with great advantages.

With the aim of exploring the source of heterogeneity, we performed sensitivity analysis and found that none of those studies changed the results greatly. Neither the Egger test nor the Begg's funnel plot revealed obvious publication bias for clinicopathological and prognostic analysis. Furthermore, no evidence of publication bias in studies for diagnostic analysis existed according to the Deeks’ funnel plot asymmetry test. Despite our reliable results, more relevant studies should be investigated to further confirm the findings of our study.

During the computerized study search, we found two previous meta‐analysis by Wang et al^4^ and Ding et al^34^ that detected the association between circRNAs and cancer. A great many differences existed among these studies. According to Wang et al, they highlighted the diagnostic value of circRNAs for human cancers especially in HCC diagnosis, whereas only five articles assessing the value of circRNAs in HCC patients were included. Ding et al assessed circRNAs as novel indicators in various tumors in fifteen articles. The pivotal role of circRNAs in HCC was not discussed. According to our study, a computerized literature search was performed and seventeen studies involving 1798 HCC patients were included. Moreover, we assessed both prognostic and diagnostic significances of circRNAs expression in HCC patients. Nevertheless, larger‐scale and higher‐quality studies conducted by multicenters were warranted to further confirm these findings.

Whereas, several limitations should be acknowledged in the present study. Firstly, because of limited number of articles, we failed to perform a subgroup analysis in terms of different circRNAs. Secondly, functional studies associated with underlying mechanisms of circRNAs in the tumorigenesis are needed. Thirdly, the relatively small number of patients might bring about the insufficiency of statistical power.^14^ Finally, several HRs outcomes were not available in the studies directly. Accordingly, HRs were extracted from the Kaplan‐Meier curves or calculated in accordance with the method of Parmar et al.^35^ However, it may introduce potential source of bias.

In conclusion, our study revealed a significant relationship between high expression of circRNAs and clinicopathological, prognostic, and diagnostic values in HCC patients. Additionally, circRNAs may be novel biomarkers and therapeutic targets for HCC. More studies are warranted to investigate the value of circRNAs in HCC patients for years to come.

## CONFLICT OF INTEREST

All authors have declared no competing interest.

## AVAILABILITY OF DATA AND MATERIALS

The datasets in the current study are available from the corresponding author on reasonable request.

## ETHICS APPROVAL AND CONSENT TO PARTICIPATE

Not applicable.
