# Supplementary figures and images for: Prognostic and diagnostic significance of circRNAs expression in hepatocellular carcinoma patients: A meta‐analysis
Source: Cancer Med. 2019 Jan 28;8(3):1148–56. doi: 10.1002/cam4.1939 (PMC6434206; doi:10.1002/cam4.1939)

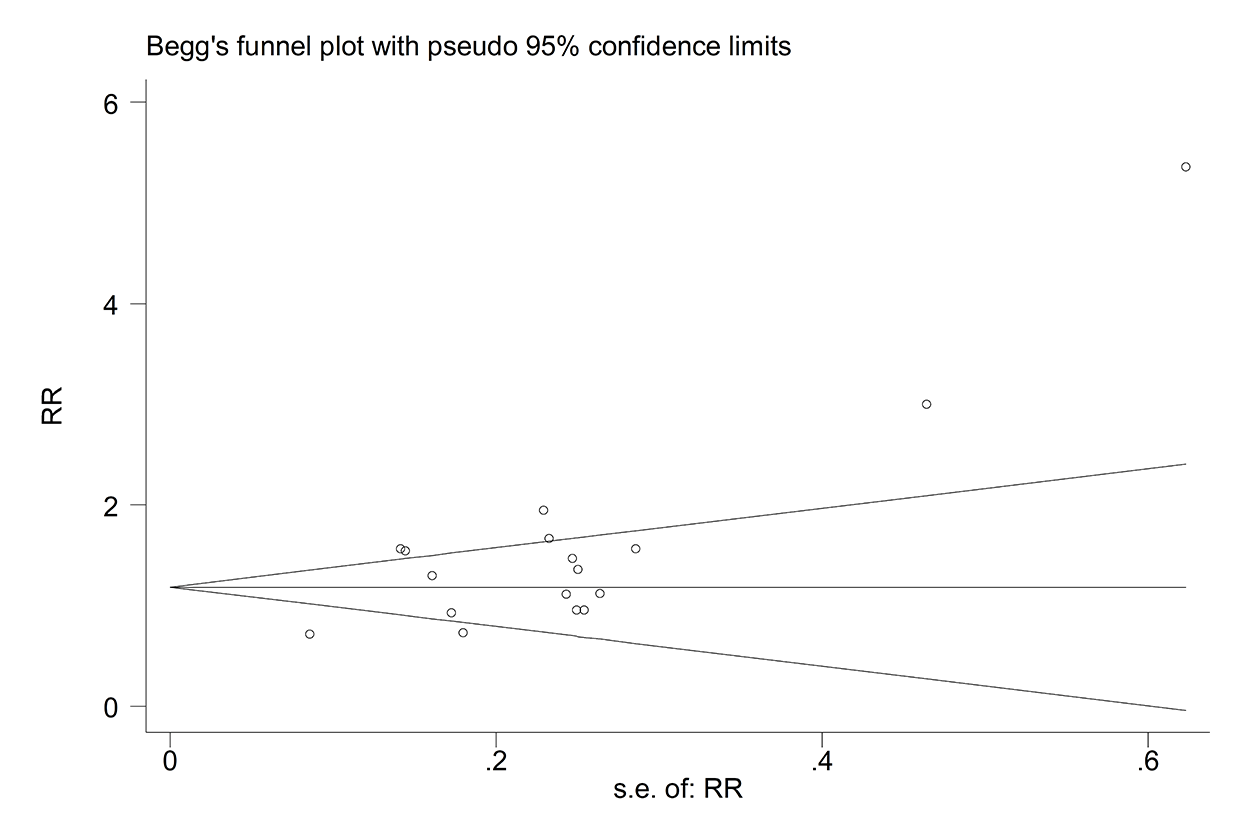

Supplement: Supplementary file 1 [file CAM4-8-1148-s001.tif]

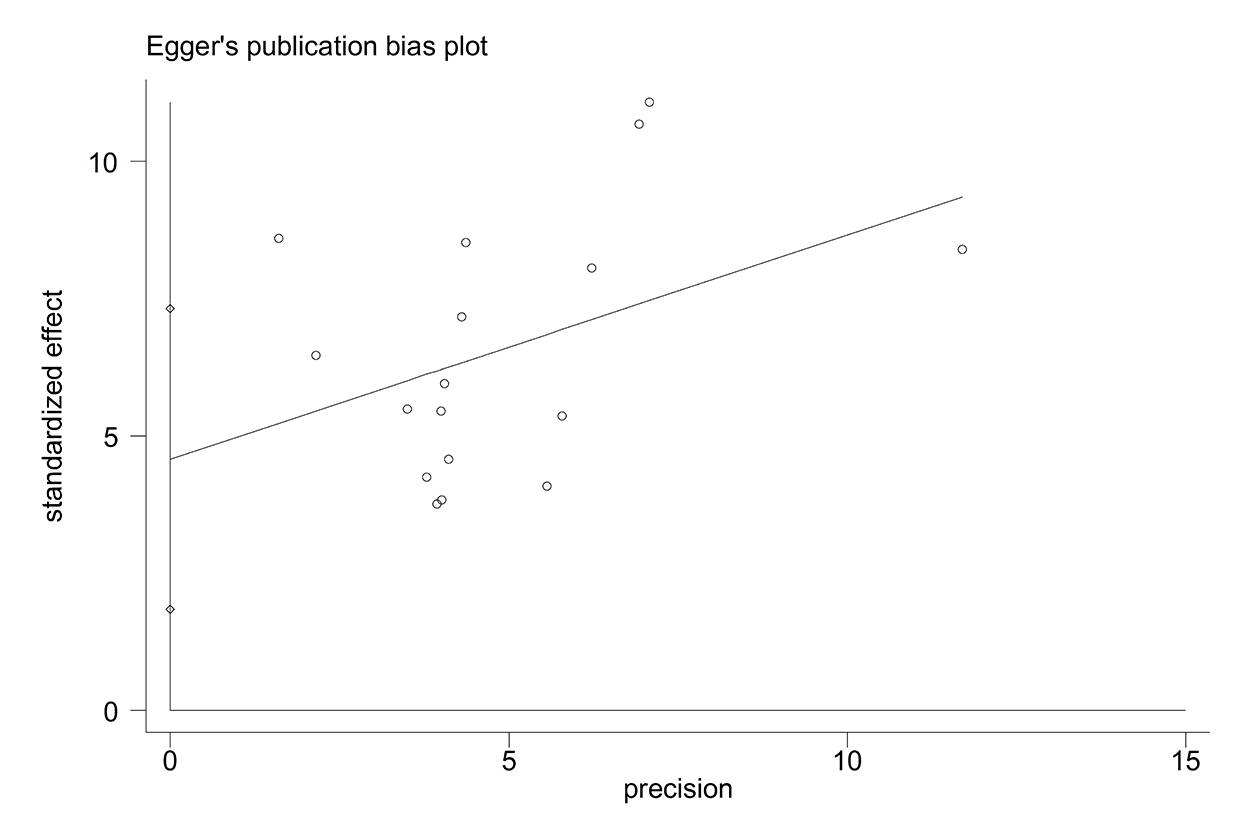

Supplement: Supplementary file 2 [file CAM4-8-1148-s002.tif]

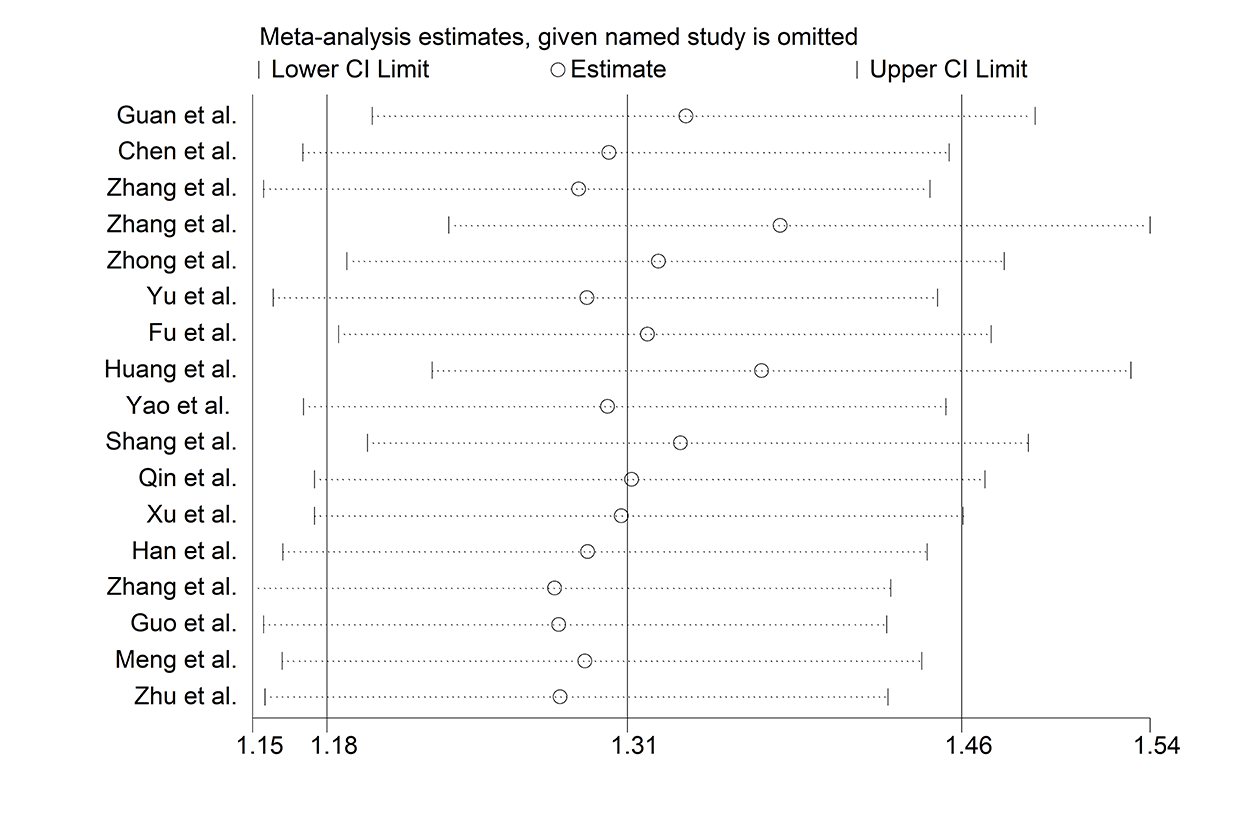

Supplement: Supplementary file 3 [file CAM4-8-1148-s003.tif]

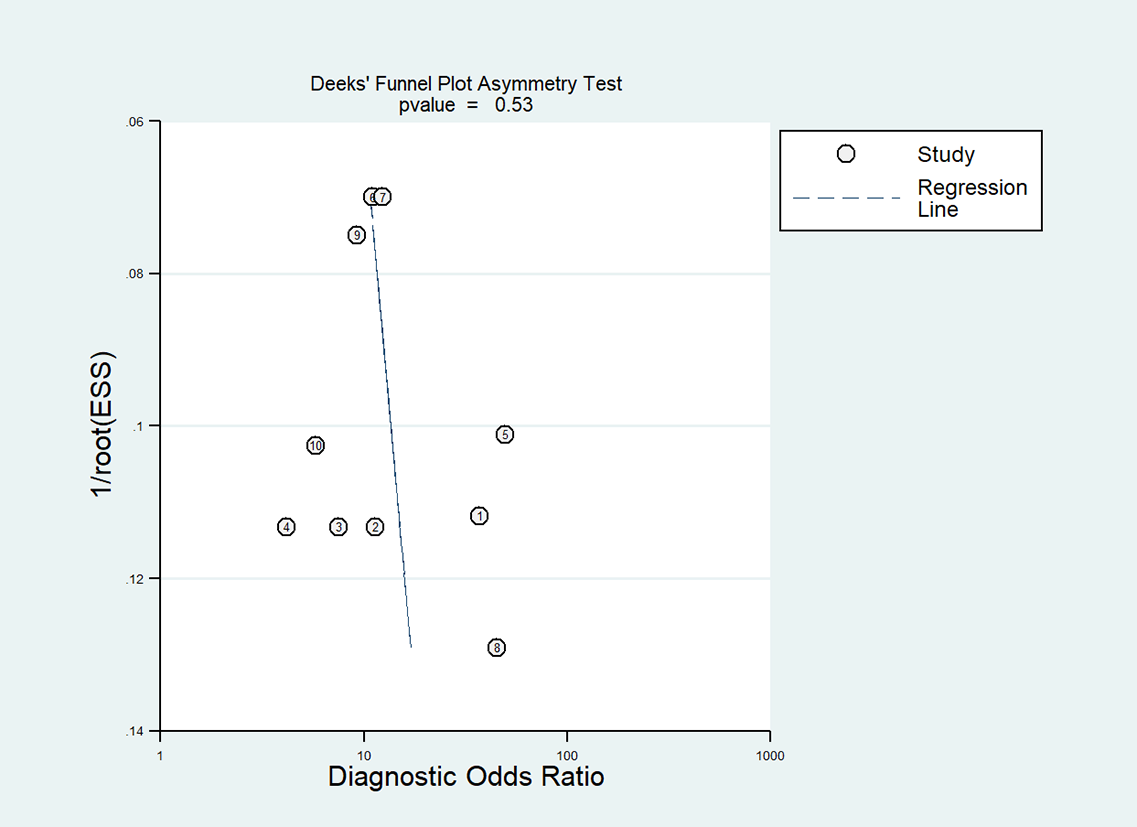

Supplement: Supplementary file 4 [file CAM4-8-1148-s004.tif]
