# Supplementary material for: Prognostic and diagnostic significance of circRNAs expression in hepatocellular carcinoma patients: A meta‐analysis
Source: Cancer Med. 2019 Jan 28;8(3):1148–56. doi: 10.1002/cam4.1939 (PMC6434206; doi:10.1002/cam4.1939)
Supplement: Supplementary file 5 [file CAM4-8-1148-s005.docx]

**Table S1.** Quality assessment of eligible studies (Newcastle-Ottawa Scale).

| **Study** | **Selection** | | | **Comparability** | | | **Outcome** | | **Total** |
| --- | --- | --- | --- | --- | --- | --- | --- | --- | --- |
|  | **Adequacy of case definition** | **Number**  **of case** | **Representativeness of the cases** | **Ascertainment of**  **relevant cancers** | **Ascertainment of**  **detection method** | **CircRNA expression** | **Assessment of outcome** | **Adequate**  **follow up** |  |
| Han et al. | 1 | 0 | 1 | 1 | 1 | 1 | 1 | 1 | 7 |
| Zhang et al. | 1 | 0 | 1 | 1 | 1 | 1 | 1 | 1 | 7 |
| Meng et al. | 1 | 0 | 1 | 1 | 1 | 1 | 1 | 1 | 7 |
| Xu et al. | 1 | 1 | 1 | 1 | 1 | 1 | 1 | 1 | 8 |
| Guo et al. | 1 | 1 | 1 | 1 | 1 | 1 | 1 | 1 | 8 |
| Zhu et al. | 1 | 1 | 1 | 1 | 1 | 1 | 1 | 1 | 8 |
| Guan et al. | 1 | 1 | 1 | 1 | 1 | 1 | 1 | 1 | 8 |
| Chen et al. | 1 | 0 | 1 | 1 | 1 | 1 | 1 | 1 | 7 |
| Zhang et al. | 1 | 0 | 1 | 1 | 1 | 1 | 1 | 1 | 7 |
| Zhang et al. | 1 | 1 | 1 | 1 | 1 | 1 | 1 | 0 | 7 |
| Zhong et al. | 1 | 1 | 1 | 1 | 1 | 1 | 1 | 1 | 8 |
| Yu et al. | 1 | 0 | 1 | 1 | 1 | 1 | 1 | 1 | 7 |
| Fu et al. | 1 | 1 | 1 | 1 | 1 | 1 | 1 | 1 | 8 |
| Huang et al. | 1 | 1 | 1 | 1 | 1 | 1 | 1 | 1 | 8 |
| Yao et al. | 1 | 1 | 1 | 0 | 1 | 1 | 1 | 1 | 7 |
| Shang et al. | 1 | 1 | 1 | 1 | 1 | 1 | 1 | 1 | 8 |
| Qin et al. | 1 | 1 | 1 | 1 | 1 | 1 | 1 | 1 | 8 |
